# The key to an effective AI-powered digital pathology: Establishing a symbiotic workflow between pathologists and machine

**DOI:** 10.1016/j.jpi.2022.100156

**Published:** 2022-11-10

**Authors:** Mohammad Hossein Jarrahi, Vahid Davoudi, Mohammad Haeri

**Affiliations:** a University of North Carolina, 100 Manning Hall, Chapel Hill, NC 27599, USA; b Alzheimer Disease Research Center, University of Kansas, Kansas University Medical Center, Kansas City, Kansas, USA; c Department of Pathology & Laboratory Medicine, University of Kansas Medical Center, Kansas City, Kansas, USA

**Keywords:** Artificial intelligence, Computational pathology, Digital pathology, Image analysis, Explainable AI, Human-in-the-loop

## Abstract

Pathology is a fundamental element of modern medicine that determines the final diagnosis of medical conditions, leads medical decisions, and portrays the prognosis. Due to continuous improvements in AI capabilities (e.g., object recognition and image processing), intelligent systems are bound to play a key role in augmenting pathology research and clinical practices. Despite the pervasive deployment of computational approaches in similar fields such as radiology, there has been less success in integrating AI in clinical practices and histopathological diagnosis. This is partly due to the opacity of end-to-end AI systems, which raises issues of interoperability and accountability of medical practices. In this article, we draw on interactive machine learning to take advantage of AI in digital pathology to open the black box of AI and generate a more effective partnership between pathologists and AI systems based on the metaphors of parameterization and implicitization.

## Introduction

The application of AI for medical diagnosis is expanding rapidly.^21^ In recent years, the use of machine learning, and specifically deep learning has made some great strides in computer-mediated pathologic diagnosis and offers promising standardized, reproducible, and reliable potentials for digital image analysis.^18^ Deep learning draws on artificial neural networks, which allow the system to develop its own logic. This provides self-learning capacities and unique affordances for “model-based assessment of routine diagnostic features in pathology, and the ability to extract and identify novel features that provide insights into a disease”.^1^ Applications of AI in routine pathology, however, are constrained by some key challenges, including infrastructural deficiencies such as limited or non-optimal digitization practices, reliable computational infrastructures, or lack of reliable data storage.^9^ A closer examination into limited applications of computer-mediated tools in pathology, particularly AI systems, reveals deeper issues than failed technology and points to practice-level dynamics.^19^

The opaque (black box) nature of AI models and how AI algorithms are integrated in a clinical diagnosis is one of the largest stumbling blocks.^1^ Most existing algorithmic systems cannot effectively reveal underlying reasons behind their decisions, making it intractable to trust AI applications in computational pathology.^20^ Even explainable AI systems (XAI) currently employed in medical settings present significant interoperability challenges.^12^ These systems do not necessarily relate to the needs and practices of end-users (medical professionals); for example, they may provide a large amount of information or in formats that appear indecipherable to physicians (e.g., the feature–importance vector format).^22^

The interoperability challenges lead to accountability issues in high-stake decisions in pathology as human experts (i.e., pathologists) are deemed irreplaceable and must actively participate in decision making. As others noted, historically “human–machine collaborations have performed better than either one alone” in these contexts,^13^ and such a partnership requires opening the black box of AI and making results transparent and explainable for different stakeholders.^8^^,^^9^ The combination of these challenges could render pathologists, regulators, and other stakeholders skeptical of the bottom-line impacts of AI in clinical workflows.^11^ In what follows, we provide an overview of the common end-to-end approach towards the application of AI in medical diagnoses and juxtapose it with our approach, which centers on explainable AI.

## End-to-end AI

**Fig. 1:**
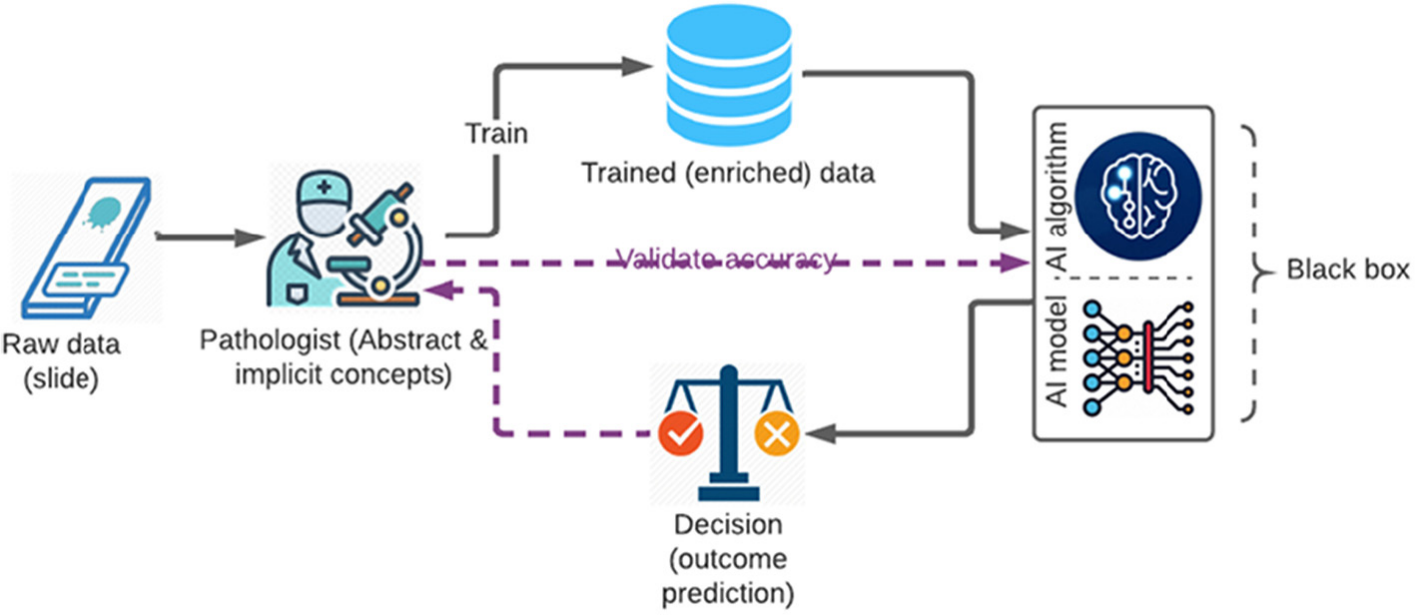
End-to-end AI work system.

Typically, deep learning relies on standard H&E and immunohistochemistry glass slides labeled by pathologists or trained experts for its initial training. The annotation can include information related to patient outcomes, clinical classifications, and image annotations. Some studies even go around the issue of pixel-wise manual annotations by pathologists, training AI models on large training data sets (e.g., whole slide images) and offering automatic extraction and identification of histopathological features (e.g., Campanella et al.^5^). In this typical work system, the role of the human expert is either eliminated or relegated to: (1) trainer of the algorithmic system, and (2) validator of the system’s recommendations and diagnostic decisions (purple lines in Fig. 1). It is important to note that beyond this stage in building and training end-to-end AI systems, the pathologist is not replaced in clinical practices but provided with automated binary decisions. Such routines of AI-pathologist work systems arguably increase the speed and efficiency since the pathologists receive the final diagnosis. However, as the AI-driven diagnoses are not evidence-based, the pathologists are not necessarily provided with reasonings for AI-generated diagnoses.^15^ Moreover, the clinicians cannot interact with machines and their logic and therefore few opportunities exist for mutual learning. Such approaches could reflect a pervasive implicit assumption among some AI developers, who treat domain experts as “non-essential” and conceive their expertise as needed only in service of building and optimizing algorithms in a utilitarian manner.^17^

## Explainable AI: Expert-in-the-loop

The ideal approach is informed and inspired by current research on interactive machine learning (interactive ML),^7^ which is focused on training and optimizing algorithms through intuitive human–computer interface, and iteratively integrating users’ (pathologist) feedback into informing histopathologic features. In interactive ML, human experts are not just labelers or annotators of pathology images, but they serve as the primary driver and guide of the machine in exploring the pathologic condition; the pathologist interacts with data and may directly contribute to feature extraction in the process of working jointly with the machine.^3^

### Application context

We recently developed a framework utilizing an explainable AI workflow for grading of meningioma, the most common primary brain tumor (see Gu et al.^6^ for more details on the user study with pathologists). Meningioma is classified into 3 grades based on its histopathological features. The prognosis, recurrence rate, and treatment management of different grades of meningioma vary significantly. There are 4 routes to diagnosis of a grade 2 meningioma including brain invasion, more than 3 mitoses per 10 high power field (HPF), and 3 out of the following 5 morphological characteristics which are hypercellularity, sheeting architecture, prominent nucleoli, spontaneous necrosis, and small cell component. The presence of 3 architectural morphologies, mentioned above, upgrades a grade 1 meningioma to a grade 2 tumor. The last route to a grade 2 meningioma is specific subtypes including clear-cell and chordoid meningiomas. The highlight of this system is the explainability of tumor grading with a language understandable for pathologists. Accordingly, this system involves a redesigned workflow with key contributions from both pathologists and AI in faster and more reliable grading of meningioma.

We articulate the symbiotic interaction between pathologists and the AI system through the continuous process of parameterization and implicitization. These 2 concepts are used as metaphors to explain the reciprocal process through which the 2 partners work together and contribute to explainable AI workflows in a pathology setting.

**Fig. 2:**
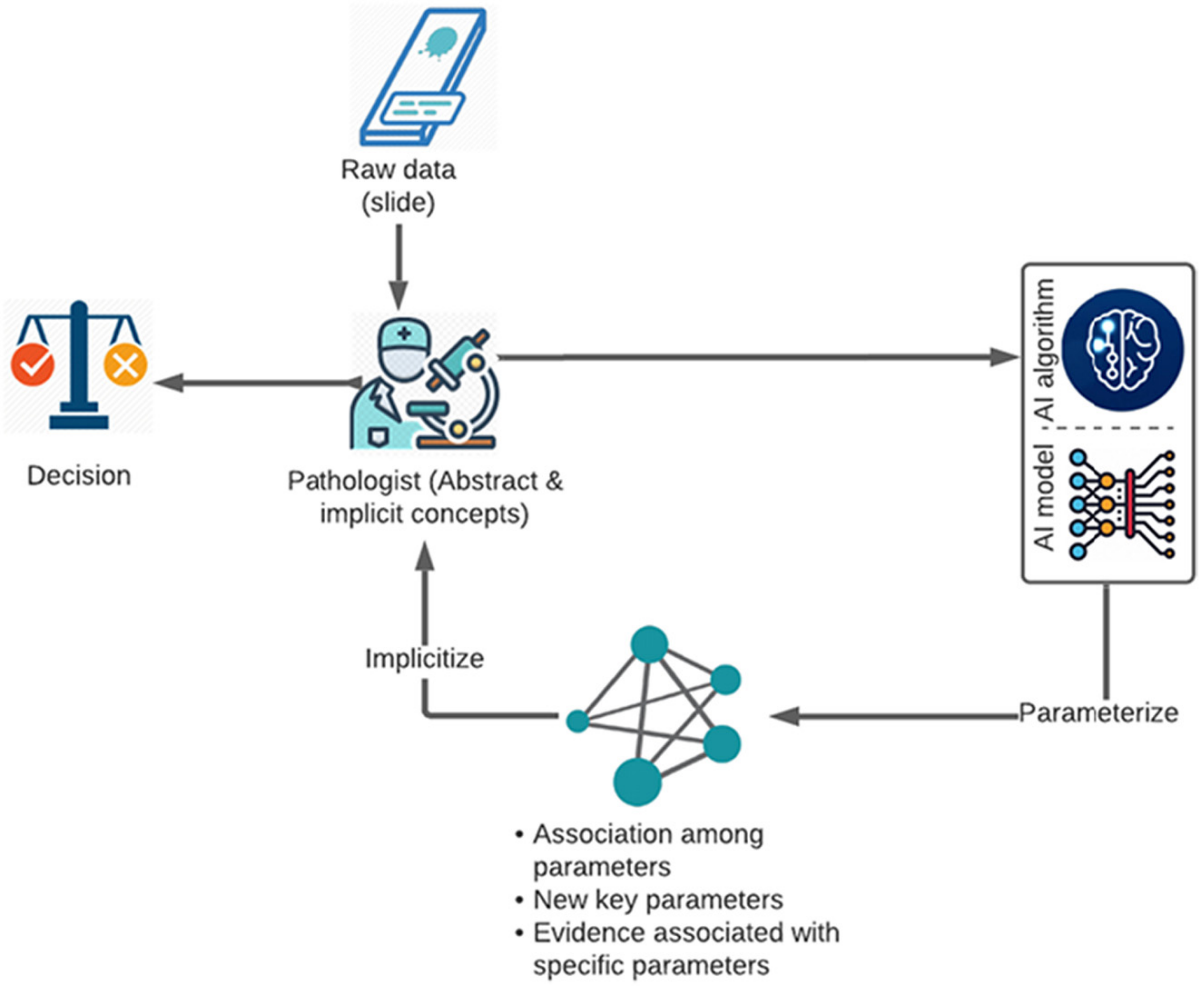
Explainable, expert-in-the-loop AI work system.

Inspired by the mathematical process of parameterization and implicitization (mostly used in geometry), we describe how an effective partnership between humans and AI can help reduce uncertainty and complexity as key factors that riddle the efficacy of decision making in clinical settings (see Fig. 2). Humans have unique capacities in dealing with uncertainty, whereas AI systems are more competent in handling the complexity of information.^10^ Parameterization and implicitization help to open the black box of AI-empowered systems and fulfill the vision of human–AI partnership. This approach reinforces the mutual learning between human experts and AI – the inner circle in Fig. 2 facilitates mutual learning through continuous parameterization and implicitization.

### Parameterization

Parameterization is the process of identifying and expressing implicit equations (e.g., a disease) using a variety of parameters (e.g., histopathological features). Parameterization is instrumental in defining the state or quality of a system (e.g., a pathologic condition). Parameterization “divides and conquers” the entire pathologic state and breaks it down into several histopathological features (parameters), so that these parameters and their associations become simple enough to be observed and understood directly.

Human experts' contributions are still irreplaceable in 2 ways: (1) pathologists define the criteria for a specific diagnosis and their pathologic and disease-specific knowledge is required to choose “good” parameters (normal features) which is always the first step in interactive ML^3^; (2) pathologists must stay “in the loop” to decide if pathologic features and relationships extracted by the machine are meaningful.^2^ AI provides 2 contributions to the parameterization process: (1) it extracts features/parameters defined by pathologists (e.g., it showcases the structure of cells in the data with nuclei larger than certain diameters as an example); (2) it could produce new medical knowledge (latent knowledge) by discovering new pathologic features and new association between parameters unnoticed, undetectable, or unknown by pathologists.^16^ Due to its analytical superiority in analyzing a large number of features and data points, AI can uncover pathologic phenotypes and associations that were not previously considered prominent. However, it is the pathologist who decides if such new features/associations discovered by AI are meaningful and valuable. The valuable new knowledge will be fed back to the algorithm for developing better feature extraction and diagnostic decisions.

In cited work of grading meningioma, the algorithm looks for all the features that are compatible with a CNS grade 2 meningioma and provides the histopathological determinants such as brain invasion, specific subtypes, and all other features that qualify a CNS grade 2 meningioma. The AI focuses on the criteria that are used by pathologists to grade the meningioma, not on a black-box process with which a pathologist has no way of interacting. The system receives feedback from pathologists based on their confirmation or rejection of an extracted feature, which leads to improved recognition of abnormal structures by the system. In addition, AI can reduce complexity by adapting to evidence-based feature extraction and diagnosis. The advantage of the system is the speed of quantification, pattern recognition in small foci and characteristics that could take hours for a pathologist to accomplish.

### Implicitization

Implicitization is the inverse process of parameterization. Implicitization refers to converting back parameters (pathologic features) and their association to a single implicit disease state (certain diagnosis). Implicitization is key in keeping the holistic nature of a pathologic state in view (the pathology has higher dimensions and details than the sums of various pathologic features and their association). In the decision-making clinical context, pathologists have a competitive advantage in generating and maintaining a holistic view, which is central in overcoming uncertainty, defined as a lack of information about the big picture and alternative decision routes.^10^ Indeed, machines have limited capacities in accounting for all pathologic presentations, their variants, past medical history, managements, prognosis, and follow-ups. The histological diagnosis, empowered by AI, offers a crucial but unavoidably limited perspective. This piece of analysis must be done by an expert pathologist who adapts a more comprehensive clinical approach to reach the final integrated diagnosis and then recommend the most appropriate next step for patient management. The broader medical context consists of many factors, and the final diagnostic report often includes information such as sub-type/variant of cancer, grade, stage, as well as prognosis and recommendations for further management/treatment decisions. AI algorithms in current forms lack this pathologist-level *clinical intelligence*, and only perform efficiently and with high accuracy in narrow and specific (and often binary) tasks of histological diagnosis. Implicitization involves a crucial sensemaking component through which pathologists put together all the parameters as well as contextual medical information and decide if a certain diagnosis or prognosis “makes sense.” This noted, AI can potentially contribute to the implicitization process by helping human experts in visualizing the associations between many parameters (i.e., patterns of associations) and understanding the ways interactions among those parameters/features, or their unique combinations, may give rise to the problem at hand. However, it is important to note that verification of the abovementioned associations requires big data-driven analysis which is the subject of longstanding discussions beyond the scope of this article.

In this workflow, AI is utilized to recognize quantified cytoarchitecture and morphology and the final decision is made by the pathologist by looking at the extracted data and other elements associated with the patient’s condition. The pathologist decides on the accuracy of the results by going over all the findings detected and presented by the AI system. Such an approach is empowered by close and transparent interaction between AI systems and pathologists. Finally, the pathologist makes the final integrated diagnosis and assembles the final report for the other health providers and clinicians.

This is a case for a pathologist-in-the loop approach. The holistic view possessed by medical experts is often of an implicit/tacit nature and derives from an intuitive decision-making style.^4^ Considering pathologist–AI interactions, pathologists can: (1) specify the key pathologic features and feed them into the AI system, and (2) put the AI-enabled parameterization into a holistic pathologic perspective to produce a final decision. A crucial component of the decision-making process (2) here relates to the pathologists' unique ability to “contextualize.” While AI systems may reveal more contextual features, it is the human expert that can bring it all together (e.g., patients' prior history) in the form of a final decision/diagnosis and report. Finally, the interpretability achieved in an “expert-in-the-loop AI work system” enables higher levels of trust and accountability as the pathologist retakes the helm in understanding how the AI system arrives at a decision in detecting a certain pathologic condition. In this new work system, each partner brings unique capabilities and comparative advantage to the table (see Table 1). Such an expert-in-loop approach also allows the pathologist to address the disconnect “between AI’s know-what and experts’ know-how” commonly manifested in applications of AI in medical settings.^14^

**Table 1:** Constitution of human experts and AI system.

Actors	Parameterization	Examples in pathology	Implicitization	Examples in pathology
Human experts (pathologists)	• Initiate the criteria for a specific feature/diagnosis. • Decide if discovered parameters and associations (by machine) are meaningful.	• Pathologists select (a) the features of ‘mitosis,’ or (b) brain invasion criteria. • Pathologists verify (a) a mitosis event detected by the system, or (b) a brain invasion.	• Bring different parameters together towards a holistic diagnosis. • Place decisions into the broader diagnostic context.	• Pathologists/clinicians decide if the criteria presented by the AI system based on the whole context of the patient (e.g., histologic features) for upgrading the tumor are met. • Pathologists make the final integrated diagnosis and assembles the final report.
AI systems	• Extract parameters defined by pathologists. • Discover new parameters impacting the case in question.	• AI searches for the features associated with a CNS grade 2 meningioma. • AI provides new histopathologic determinants previously not obvious to pathologists.	• Help identify the relationships among parameters and their interactions.	• AI identifies quantified cytoarchitecture and morphology associated with a specific disease.

In practice, experts address this uncertainty by drawing on rich know-how practices, which were not incorporated into these ML-based tools.

## Conclusion

Lack of interpretability and transparency stands in the way of clinical adoptions of many AI systems.^9^ End-to-end AI systems gloss over the complicated contexts of clinical decision-making in pathology, posing regulatory and pragmatic challenges. Rising evidence suggests even the explainability features recently added to the end-to-end AI system as post hoc interpretations appear ineffective in real-world clinical practices.^12^ The symbiotic relationship presented here in the form of the expert-in-the-loop AI work system could clarify the unique contributions of both humans and machines in a mutual workflow and raise trust in the application of AI in a pathology setting. Our approach takes up the challenge of interpretability and accountability, as common end-to-end approaches based on artificial neural networks with their black box “do not provide a verifiable path to understanding the rationale behind its decisions”.^19^

## Acknowledgments and Disclosure of Funding

We appreciate Hongyan Gu, Yifan Xu, and Xiang 'Anthony' Chen for their contribution to this research project.

## Declaration of interests

The authors declare that they have no known competing financial interests or personal relationships that could have appeared to influence the work reported in this paper.

We do not have any competing financial interest or personal relationships that might have influenced this work; and there are not financial interests involved in developing or publishing this work.
